# Sitafloxacin Expresses Potent Anti-*Mycobacterium abscessus* Activity

**DOI:** 10.3389/fmicb.2021.779531

**Published:** 2022-01-06

**Authors:** Siyuan He, Qi Guo, Lan Zhao, Liyun Xu, Junsheng Fan, Wenye Wu, Zhemin Zhang, Bing Li, Haiqing Chu

**Affiliations:** ^1^Department of Respiratory and Critical Care Medicine, Shanghai Pulmonary Hospital, School of Medicine, Tongji University, Shanghai, China; ^2^School of Medicine, Tongji University, Shanghai, China; ^3^Shanghai Key Laboratory of Tuberculosis, Shanghai Pulmonary Hospital, School of Medicine, Tongji University, Shanghai, China

**Keywords:** *Mycobacterium abscessus*, *in vitro*, intracellular, quinolone, sitafloxacin

## Abstract

Therapeutic options for treating *Mycobacterium abscessus* infections are extremely limited; quinolones are important. The *in vitro* anti-*M. abscessus* activities of nine quinolones, emphasizing sitafloxacin, were investigated. Antimicrobial susceptibility testing was performed on 10 non-tuberculous mycobacterium reference strains and 194 clinical, *M. abscessus* isolates. The activity of sitafloxacin against intracellular *M. abscessus* residing within macrophages was also evaluated. A checkerboard assay was conducted to determine synergy between sitafloxacin and 10 clinically important antibiotics. Among the nine quinolones tested, sitafloxacin exhibited the greatest anti-*M. abscessus* activity with MIC_50_ and MIC_90_ of 1 and 2 mg/L, respectively. Sitafloxacin exerted a bacteriostatic effect on *M. abscessus* and inhibited the intracellular growth of *M. abscessus* at concentrations equivalent to clarithromycin. No antagonism between sitafloxacin and 10 clinically important anti-*M. abscessus* antibiotics was evident. In summary, sitafloxacin exhibited a significant advantage relative to other quinolones in inhibiting the growth of *M. abscessus in vitro*, suggesting the potential inclusion of sitafloxacin in new strategies to treat *M. abscessus* infections.

## Introduction

The number of infections caused by non-tuberculous mycobacteria (NTM) is increasing globally (Cowman et al., 2019; Johansen et al., 2020; Mourad et al., 2021). *Mycobacterium abscessus* is an important pathogenic NTM for patients with bronchiectasis, chronic obstructive pulmonary disease and cystic fibrosis. *M. abscessus* infections are the most challenging to treat due to an intrinsic resistance to many common antibiotics (Fletcher et al., 2016; Degiacomi et al., 2019). No evidence-based antibiotic regimen has been established to date; the cure rate and rate of recurrence are far from satisfactory (Stout et al., 2016; Haworth et al., 2017; Daley et al., 2020). Consequently, there is an urgent need for new therapeutic options.

Sitafloxacin is an oral quinolone, which exhibits excellent antibacterial activity by simultaneously inhibiting DNA gyrase and topoisomerase IV in a broad range of bacteria including mycobacteria (Tomioka et al., 1999; Sato et al., 2003; Amano et al., 2016; Nakajima et al., 2016; Asakura et al., 2019; Kamada et al., 2021). Reports of the successful treatment of cases of *M. abscessus*-associated pneumonia with drug combinations that included sitafloxacin have provoked a recent interest in using sitafloxacin for treating *M. abscessus* infections (Oka et al., 2021; Takano et al., 2021). Information concerning the anti-*M. abscessus* activity of sitafloxacin is limited, however.

A systematic evaluation of the antimicrobial activity of sitafloxacin against a large number of clinical, *M. abscessus* isolates was undertaken for the first time in the present study. Sitafloxacin was active against *M. abscessus* growing *in vitro*; sitafloxacin exhibited the lowest minimum inhibitory concentration (MIC) among nine quinolones tested. Importantly, sitafloxacin was especially effective in inhibiting the growth of organisms growing intracellularly, i.e., in macrophages. Sitafloxacin did not antagonize the activity of antibiotics most frequently used to treat *M. abscessus* infections. These findings demonstrate the significant advantages of sitafloxacin over other quinolones, which may provide a new approach to treating *M. abscessus* infections.

## Materials and Methods

### Bacterial Strains

A total of 10 NTM reference strains and 194 clinical, *M. abscessus* isolates were evaluated. The following reference strains: *M. abscessus* subsp. *abscessus* (ATCC 19977), *M. avium* (ATCC 25291), *M. intracellulare* (ATCC 13950), *M. kansasii* (ATCC 12478), *M. fortuitum* (ATCC 6841), *M. gordonae* (ATCC 14470), *M. scrofulaceum* (ATCC19981), *M. peregrinum* (ATCC700686), and *M. xenopi* (ATCC19250) were purchased from the American Type Culture Collection (ATCC; VA, United States). *M. abscessus* subsp. *massiliense* (CIP108297) was purchased from the Biological Resource Center of Institut Pasteur (CIP; Paris, France). Detailed information regarding the clinical isolates was provided in a previous study (Guo et al., 2020). All strains were grown at 37°C on Middlebrook 7H10 agar plates supplemented with 10% OADC and 0.2% glycerol, or with continuous shaking in Middlebrook 7H9 broth supplemented with 10% OADC and 0.05% Tween 80. Middlebrook 7H9 broth, M7H10 agar, cation-adjusted Mueller-Hinton II broth and OADC were purchased from Becton Dickinson and Company (NJ, United States).

### Antimicrobial Agents

Clarithromycin, azithromycin, amikacin, cefoxitin, imipenem, tigecycline, linezolid, and ciprofloxacin were purchased from Sigma-Aldrich Company (St. Louis, MO, United States). Bedaquiline was purchased from Biopharmaleader (Biopharmaleader, China). Nemonoxacin was purchased from Zhejiang Pharmaceutical Co., Ltd. (Xinchang Pharmaceutical Factory, Zhejiang, China). All remaining antibiotics were purchased from MCE (MedChemExpress, Monmouth Junction, NJ, United States). Sitafloxacin, clarithromycin, clofazimine, bedaquiline, rifabutin and sparfloxacin were solubilized in 100% DMSO; levofloxacin and nemonoxacin were dissolved in 1%NAOH; azithromycin was dissolved in absolute ethanol; and the remaining antibiotics were prepared in de-ionized water. The antibiotics were aliquoted, stored at −20°C and serially diluted just prior to experimental use.

### Minimum Inhibitory Concentration and Minimum Bactericidal Concentration Determination

Antibiotic susceptibility was determined by the broth microdilution method according to Clinical and Laboratory Standards Institute (CLSI) document M24-A2 (Woods et al., 2011). Briefly, individual *M. abscessus* colonies were picked from M7H10 agar plates, grown to logarithmic phase in M7H9 broth, diluted to McFarland 0.5 with sterile saline, and adjusted to 1 × 10^5^ to 5 × 10^5^ CFU/ml in cation-adjusted Mueller-Hinton broth (CAMHB). Nine quinolones (ciprofloxacin, levofloxacin, moxifloxacin, nemonoxacin, sitafloxacin, gatifloxacin, delafloxacin, garenoxacin, and sparfloxacin) were tested. Antibiotics were serially diluted 1:2 in a 96-well microtiter plate (working concentrations ranged from 0.25 to 64 mg/L), and 100 μl of bacteria suspended in CAMHB was added to each well. The microtiter plates were sealed with parafilm and rapid growing mycobacteria were incubated for 3–5 days at 37°C in ambient air. Slow growing mycobacteria were incubated for 7–10 days, or the time it took for the control wells without antibiotics to exhibit visible growth. MIC was defined as the minimum drug concentration at which no visual bacterial growth occurred (Supplementary Figure 1).

*M. abscessus* subsp. *abscessus* ATCC 19977 and subsp. *massiliense* CIP 108297 were used in assays to determine the MBC of the following seven quinolones: sitafloxacin, ciprofloxacin, levofloxacin, moxifloxacin, nemonoxacin, gatifloxacin, and sparfloxacin. Delafloxacin and garenoxacin were not included in this analysis due to their high MIC values. The contents of each microtiter plate well containing a drug concentration greater than the MIC were evenly suspended on day 4 following completion of the antimicrobial susceptibility test. One hundred microliter aliquots of each well were cultured on Middlebrook 7 H10 supplemented with 0.2% glycerol and OADC enrichment. CFUs were quantified after an additional 5 days incubation at 37°C. The MBC values were defined as the minimum drug concentration that prevented 99.9% bacterial growth expressed in CFU/ml. An antibiotic was considered bactericidal if the MBC/MIC ratio was ≤ 4, or bacteriostatic if the ratio was > 4.

### Anti-mycobacterial Drug Synergy

Synergy between sitafloxacin and clarithromycin, azithromycin, amikacin, linezolid, clofazimine, imipenem, tigecycline, bedaquiline, cefoxitin, and rifabutin was assessed *in vitro* using the broth microdilution chequerboard titration technique as previously described (Kaushik et al., 2017). *M. abscessus* subsp. *abscessus* reference strain ATCC 19977, *M. abscessus* subsp. *massiliense* reference strain CIP 108297, and six clinical isolates (three subsp. *abscessus* and three subsp. *massiliense*) were used for evaluation. Synergy test results were interpreted based upon the fractional inhibitory concentration index (FICI), calculated using the following formula: FICI = (MIC of antibiotic A in combination/MIC of antibiotic A alone) + (MIC of sitafloxacin in the combination/MIC of sitafloxacin alone). Drug interactions were classified as: synergy (FICI ≤ 0.5), indifference (0.5 < FICI ≤ 4) and antagonism (FICI > 4.0).

### Intracellular Killing Assay

Mouse peritoneal macrophages, frequently used to examine the factors that affect the intracellular replication of mycobacterium species, were obtained by methods previous described by us (Zhang et al., 2020). The cells were infected with *M. abscessus* (multiplicity of infection = 5) suspended in RPMI 1,640 medium supplemented with 10% fetal bovine serum (FBS). After 4 h incubation at 37°C in 5% CO2, the cells were washed three times with warm phosphate-buffered saline to remove the extracellular organisms. Fresh RPMI 1,640 medium with 10% FBS and sitafloxacin, moxifloxacin and clarithromycin at 0.2, 1, 5, and 10 μg/ml was then added; infected control cells were treated with medium and 10% FBS alone. The cells were lysed with 0.05% sodium dodecyl sulfate at 4, 24, or 48 h postinfection and the CFUs were quantified by plating serial dilutions of lysates on 7H10 agar plates. Cell viability was evaluated by trypan blue exclusion before and after infection or drug treatment at each time point.

### Statistical Analysis

Statistical differences between study groups were determined with Mann-Whitney *U*- test and Student’s non-parametric test; *P* < 0.05 was considered significant. Computations were performed using GraphPad Prism 8 (GraphPad Software, San Diego, CA).

## Results

### Sitafloxacin Expresses Superior Anti-*M. abscessus* Activity

A total of 194 clinical, *M. abscessus* isolates (148 subsp. *abscessus* and 46 subsp. *massiliense*) were collected. Compared to eight other quinolones, sitafloxacin exhibited the greatest *in vitro* activity; the MIC ranged from 0.25 to 4 mg/L, the MIC_50_ and MIC_90_ were 1 and 2 mg/L, respectively (Table 1). Subspecies analysis found that *M. abscessus* subsp. *abscessus* isolates were significantly more sensitive to sitafloxacin than *M. abscessus* subsp. *massiliense* isolates (Table 2). The minimum bactericidal concentration (MBC)/MIC ratio evidenced the bacteriostatic activity expressed by sitafloxacin toward *M. abscessus*, which is the same as that exhibited by other quinolones (Supplementary Table 1). Sitafloxacin also exhibited high activity toward rapidly growing (i.e., *M. fortuitum* and *M. peregrinum*), as well as slowly growing (i.e., *M. intracellulare, M. avium, M. kansasii, M. szulgai, M. xenopi*, and *M. scrofulaceum*), NTM reference strains (Table 3).

**TABLE 1:** MIC of 9 quinolones for 194 clinical *M. abscessus* isolates.

Antibiotics	MIC range (mg/L)	Number of isolates exhibiting the MIC (mg/L) of quinolones indicated										MIC_50_ (mg/L)^a^	MIC_90_ (mg/L)^a^
		0.25	0.5	1	2	4	8	16	32	64	>64		
Sitafloxacin	0.25–4	12	66	80	31	4	1	0	0	0	0	1	2
Ciprofloxacin	1– > 64	0	0	1	8	58	69	29	12	14	3	8	32
Levofloxacin	2– > 64	0	0	0	1	6	39	62	50	28	8	16	64
Moxifloxacin	0.5–16	0	1	10	41	76	49	14	3	0	0	4	8
Nemonoxacin	1–32	0	0	8	30	68	55	26	6	0	1	4	16
Gatifloxacin	1–32	0	0	5	27	83	50	25	5	0	0	4	16
Delafloxacin	32– > 64	0	0	0	0	0	0	0	3	23	168	>64	>64
Garenoxacin	32– > 64	0	0	0	0	0	0	0	1	18	168	>64	>64
Sparfloxacin	1– > 64	0	0	2	6	10	28	70	44	26	8	16	64

*^a^MIC_50_ and MIC_90_: the concentrations at which 50 and 90% of the clinical isolates tested, respectively, were inhibited.*

**TABLE 2:** MIC distribution of sitafloxacin for 194 clinical, *M. abscessus* isolates.

*M. abscessus*	No. of isolates	Number of isolates exhibiting the MIC (mg/L) of sitafloxacin indicated						MIC_50_ (mg/L)^a^	MIC_90_ (mg/L)*^a^*
		0.25	0.5	1	2	4	8		
subsp. *abscessus*^b^	148	11	55	63	15	3	1	1	2
subsp. *Massiliense*	46	1	10	18	16	1	0	1	2

*^a^MIC_50_ and MIC_90_, the sitafloxacin concentration at which 50 and 90% of the clinical isolates tested, respectively, were inhibited. ^b^M. abscessus subsp. abscessus was significantly more sensitive than subsp. massiliense to sitafloxacin treatment, P < 0.001.*

**TABLE 3:** MIC of 9 quinolones for NTM reference strains.

Species	STFX^a^	CIP	LFX	MFX	NOX	GXT	DLX	GAX	SPX
*M. abscessus*	0.5	4	16	4	4	4	64	>64	8
*M. massiliense*	1	8	16	4	4	8	>64	>64	32
*M. intracellulare*	2	16	16	2	32	8	32	32	4
*M. avium*	0.5	8	8	1	8	4	4	8	1
*M. kansasii*	1	4	0.5	0.125	64	0.5	4	2	0.25
*M. szulgai*	1	2	1	0.06	16	0.5	4	2	0.25
*M. scrofulaceum*	<0.125	0.5	2	0.25	1	0.5	2	1	0.125
*M. fortuitum*	0.06	0.25	0.25	0.06	0.5	0.125	1	0.5	0.125
*M. peregrinum*	<0.06	0.125	0.25	<0.06	0.25	0.125	0.5	0.25	0.125
*M. xenopi*	<0.06	<0.06	0.125	<0.06	<0.06	<0.06	0.25	0.25	<0.06

*^a^STFX, sitafloxacin; CIP, ciprofloxacin; LFX, levofloxacin; MFX, moxifloxacin; NOX, nemonoxacin; GXT, gatifloxacin; DLX, delafloxacin; GAX, garenoxacin; SPX, sparfloxacin.*

### Sitafloxacin Inhibits Intracellular *M. abscessus* Growth

**FIGURE 1:**
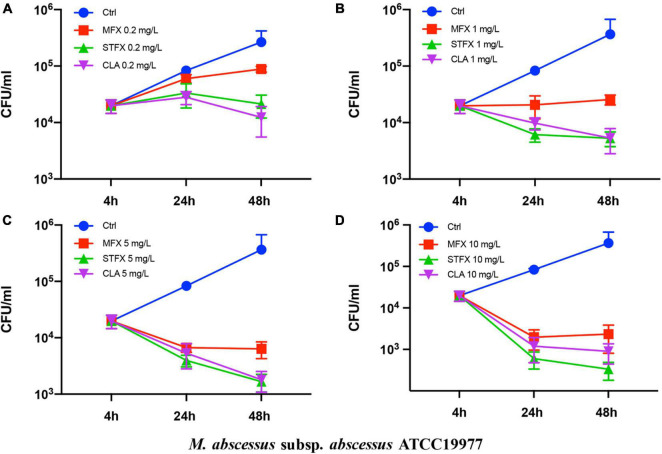
Antimicrobial activities of sitafloxacin, moxifloxacin and clarithromycin against *M. abscessus* subsp. *abscessus* ATCC19977 growing intracellularly. Antibiotics at 0.2 **(A)**, 1 **(B)**, 5 **(C)**, and10 **(D)** mg/L final concentrations were added to cultures of *M. abscessus*-infected macrophages. CFUs were quantified after 4, 24, and 48 h incubation. Ctrl, control; STFX, sitafloxacin; MFX, moxifloxacin; CLA, clarithromycin.

**FIGURE 2:**
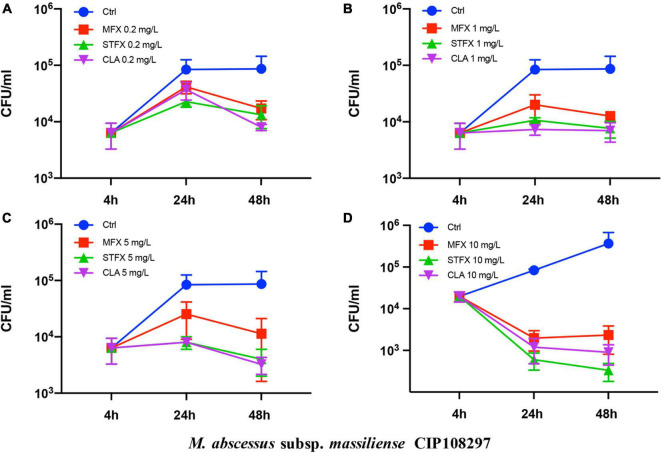
Antimicrobial activities of sitafloxacin, moxifloxacin and clarithromycin against intracellular *M. abscessus* subsp. *massiliense* CIP 108297. Antibiotics at 0.2 **(A)**, 1 **(B)**, 5 **(C)**, and10 **(D)** mg/L final concentrations were added to cultures of *M. abscessus*-infected macrophages. Bacterial CFUs were quantified after drug-treated and control *M. abscessus*-infected cells were cultured for 4, 24, and 48 h. Ctrl, control; STFX, sitafloxacin; MFX, moxifloxacin; CLA, clarithromycin.

The effects of sitafloxacin, moxifloxacin and clarithromycin on the survival of *M. abscessus* subsp. *abscessus* ATCC 19977 and subsp. *massiliense* CIP 108297 residing in primary murine peritoneal macrophages were evaluated (Figures 1, 2). Sitafloxacin, moxifloxacin, and clarithromycin inhibited the intracellular growth of *M. abscessus* for both subspecies tested; inhibition was dose-dependent. Sitafloxacin appeared to exert a greater effect than moxifloxacin at most concentrations, albeit no statistical difference was observed. The effect of sitafloxacin on the intracellular growth of *M. abscessus* subspecies was comparable to that found for clarithromycin.

### The Indifferent Interaction Between Sitafloxacin and 10 Clinically Important Anti-*M. abscessus* Antibiotics

Synergy between sitafloxacin and 10 antibiotics frequently used clinically to treat *M. abscessus* infections was tested using two reference strains and six clinical isolates. Sitafloxacin did not antagonize any of the other antibiotics tested; all interactions were indifferent [fractional inhibitory concentration index (FICI) = 0.75–2] (Table 4).

**TABLE 4:** Interaction of sitafloxacin with antibiotics frequently used to treat clinical *M. abscessus* infections.

Isolate	Subspecies	FICI^a^									
		STFX + CLA^b^	STFX + AZM	STFX + AMK	STFX + LZD	STFX + CFZ	STFX + IPM	STFX + TGC	STFX + BDQ	STFX + FOX	STFX + RFB
156	*abscessus*	1	0.75	1	1	1.5	2	2	2	1.5	2
204	*abscessus*	0.75	1	1.5	1	2	1.5	2	2	2	1.5
206	*abscessus*	1	1	1.5	1.5	2	1.5	1.5	1	1.5	1.25
98	*massiliense*	0.75	1	1	1.5	1.5	1	1.5	2	2	1.5
163	*massiliense*	1	0.75	2	1.5	2	2	1.5	2	2	1
220	*massiliense*	1	1	1	1.5	2	1.5	1.5	1.5	2	1.5
ATCC 19977	*abscessus*	0.75	1	1	1	1.5	1	1.5	2	1.25	1
CIP 108297	*massiliense*	0.75	0.75	1.25	1.5	1	1	1.5	2	1.5	1

*^a^FICI (fractional inhibitory concentration index), [(MIC of STFX in combination/MIC of STFX alone) + (MIC of second antibiotic in combination/MIC of second antibiotic alone)]. Synergy, FICI ≤ 0.5; indifference, FICI between 0.5 and ≤ 4; antagonism, FICI > 4. ^b^STFX, sitafloxacin; CLA, clarithromycin; AZM, azithromycin; AMK, amikacin; LZD, linezolid; CFZ, clofazimine; IPM, imipenem; TGC, tigecycline; BDQ, bedaquiline; FOX, cefoxitin; RFB, rifabutin.*

## Discussion

The antibacterial activity of quinolones (e.g., moxifloxacin and levofloxacin) commonly used to treat *M. abscessus* infections is unsatisfactory (Choi et al., 2012a). Sitafloxacin, a new fluoroquinolone that is effective against a broad range of bacteria, has the advantages of oral administration and superior safety (Wu et al., 2014; Amano et al., 2016; Nakajima et al., 2016; Miyazaki et al., 2019; Mori et al., 2020; Li et al., 2021a,b). Importantly, sitafloxacin exhibits antibacterial activity against *M. tuberculosis* and *M. avium* both *in vitro* and *in vivo* (Tomioka et al., 1999; Sato et al., 2003; Asakura et al., 2019). Cases of *M. abscessus* infection successfully treated with a drug combination that included sitafloxacin were reported (Oka et al., 2021; Takano et al., 2021). The efficacy of sitafloxacin alone in treating *M. abscessus* infections, however, remains to be evaluated.

Antibiotic susceptibility testing of 9 quinolones, including sitafloxacin, was conducted on 194 clinical, *M. abscessus* isolates in the present study. Sitafloxacin exhibited the lowest MIC ranging from 0.25 to 4 mg/L, and MIC_50_ and MIC_90_ of 1 and 2 mg/L, respectively. Kamada K. and coworkers reported similar results: the MIC of sitafloxacin for *M. abscessus* ranged from 0.25 to > 4 mg/L, with MIC_50_ = 1 mg/L and MIC_90_ = 4 mg/L (Kamada et al., 2021). However, other investigators determined the MIC_50_ and MIC_90_ of sitafloxacin against *M. abscessus* to be 3.13 and 6.25 mg/L, respectively, though their sample size was small (Saito et al., 1994).

A phase I clinical trial investigating the pharmacokinetics and tolerance to sitafloxacin found that a serum concentration of 1 mg/L was safely attained and well tolerated by healthy male volunteers administered a single 100 mg dose orally (Li et al., 2021b). This serum concentration is similar to the MIC_50_ of sitafloxacin for *M. abscessus* found in the current study, thus a safe and effective dose can be achieved for treating *M. abscessus* clinically. The antibacterial activity of sitafloxacin against other NTM reported here was superior or equal to that of other quinolones, which is consistent with the results of previous studies of other investigators (Saito et al., 1994; Tomioka et al., 1999; Asakura et al., 2019; Kamada et al., 2021). Notably, sitafloxacin exhibited an average MIC of 1.24 mg/L for isolates that were resistant to moxifloxacin (MIC ≥4 mg/L) in the study presented here (Supplementary Table 2). These results highlight the efficacy of sitafloxacin in treating NTM infections.

*M. abscessus* is phagocytized and replicates within macrophages thus circumventing host defenses during infection. The ability of an antibiotic to kill intracellular *M. abscessus* is essential for treatment. Moxifloxacin (the most effective, available quinolone) and clarithromycin (the current cornerstone drug for *M. abscessus* treatment) were selected and compared to sitafloxacin in an intracellular bactericidal assay. Sitafloxacin exhibited intracellular anti-*M. abscessus* (subsp. *abscessus* and subsp. *massiliense*) activity that was comparable to clarithromycin, and which tended to be greater than moxifloxacin albeit without reaching statistical significance. Notably, five severe *M. abscessus* cases recently treated with a combination of clarithromycin and sitafloxacin achieved good clinical and microbiological outcomes (Takano et al., 2021).

Multidrug combinations are required for treatment of *M. abscessus* infections (Alangaden and Lerner, 1997; Haworth et al., 2017; Daley et al., 2020). Sitafloxacin is an attractive therapeutic option though optimal effectiveness may necessitate its incorporation into a multidrug treatment plan. In the current study, no antagonism was observed between sitafloxacin and 10 clinically important antibiotics that are often used to treat *M. abscessus* infections. This suggests that sitafloxacin could easily be integrated into current anti-*M. abscessus* drug combinations. A greater number of sitafloxacin susceptible *M. abscessus* subsp. *abscessus* than *M. abscessus* subsp. *massiliense* isolates is of particular interest since infections caused by the former are more difficult to treat and exhibit a worse prognosis due to the induction of clarithromycin resistance (Koh et al., 2011; Choi et al., 2012b). Additional studies addressing this issue are envisioned.

All clinical isolates used in this study were obtained from a single sentinel hospital that receives NTM cases from throughout China. The derivation of all isolates from this single source and a potential lack of genetic diversity represent shortcomings of this study. Nonetheless, sitafloxacin expresses a high level of anti-*M. abscessus* activity *in vitro*. Moreover, sitafloxacin is compatible with other antibiotics most frequently used to treat *M. abscessus* infections. As such, sitafloxacin is a potential candidate to include in novel therapeutic anti-*M. abscessus* regimens.

## Data Availability Statement

The original contributions presented in the study are included in the article/Supplementary Material, further inquiries can be directed to the corresponding author/s.

## Author Contributions

HC, BL, SH, QG, LZ, and ZZ conceived and designed the work. LX, JF, and QG collected the bacterial isolates and compiled the data. SH and QG performed the experiments. WW, BL, and JF analyzed and interpreted the data. QG and WW were responsible for preparing the figures in the manuscript. SH, QG, and BL wrote the manuscript, which was reviewed, edited, and approved by all authors.

## Conflict of Interest

The authors declare that the research was conducted in the absence of any commercial or financial relationships that could be construed as a potential conflict of interest.

## Publisher’s Note

All claims expressed in this article are solely those of the authors and do not necessarily represent those of their affiliated organizations, or those of the publisher, the editors and the reviewers. Any product that may be evaluated in this article, or claim that may be made by its manufacturer, is not guaranteed or endorsed by the publisher.
