# Supplementary material for: Sitafloxacin Expresses Potent Anti-Mycobacterium abscessus Activity
Source: Front Microbiol. 2022 Jan 6;12:779531. doi: 10.3389/fmicb.2021.779531 (PMC8770805; doi:10.3389/fmicb.2021.779531)
Supplement: Supplementary file 1 [file Data_Sheet_1.docx]

**TABLE 1|** MBC values and anti-*M. abscessus* activity of 7 quinolones

| **Antibiotics** | **subsp. *abscessus* ATCC19977** | | | **subsp. *massiliense* CIP 108297** | | | |
| --- | --- | --- | --- | --- | --- | --- | --- |
|  | **MIC (mg/L)** | **MBC (mg/L)** | **MBC/MIC ratio** | | **MIC (mg/L)** | **MBC (mg/L)** | **MBC/MIC ratio** |
| sitafloxacin | 0.5 | 16 | 32 | | 1 | 32 | 32 |
| ciprofloxacin | 4.0 | 32 | 8 | | 8 | 64 | 8 |
| levofloxacin | 16.0 | 128 | 8 | | 16 | 128 | 8 |
| moxifloxacin | 4.0 | 64 | 16 | | 4 | 32 | 8 |
| nemonoxacin | 4.0 | 128 | 32 | | 4 | 64 | 16 |
| gatifloxacin | 4.0 | 64 | 16 | | 8 | 64 | 8 |
| sparfloxacin | 8.0 | 128 | 16 | | 32 | 128 | 4 |

**TABLE 2|** The average MIC of sitafloxacin among moxifloxacin-resistant *M. abscessus* isolates

| **Antibiotics** | **No. of isolates** | **MIC range (mg/L)** | **Average MIC (mg/L)** |
| --- | --- | --- | --- |
| sitafloxacin | 142 | 0.5-8 | 1.24 |
| moxifloxacin |  | 4-32 | 7.15 |

**
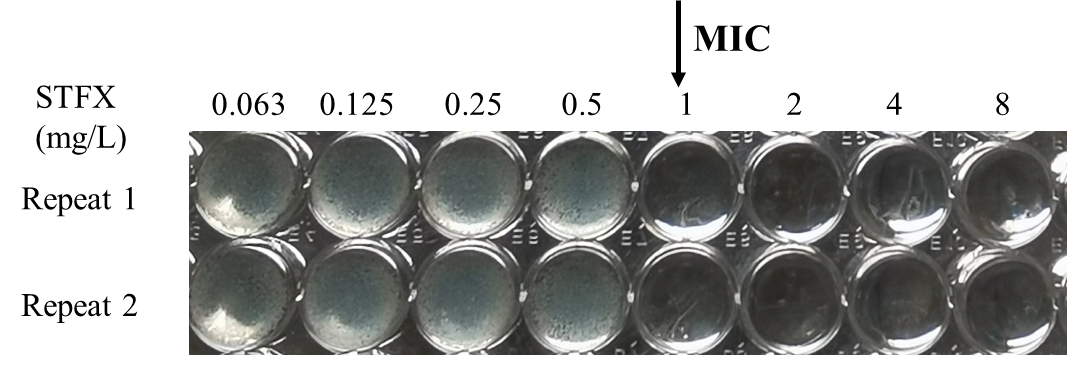
**

**Supplementary Figure 1 |** Interpretation of broth microdilution assay. The black arrow indicates the minimum inhibitory concentration (MIC), which is the lowest concentration of drug that inhibits visible growth. STFX: sitafloxacin.
